# The impact of laboratory staff training workshops on coagulation specimen rejection rates

**DOI:** 10.1371/journal.pone.0268764

**Published:** 2022-06-03

**Authors:** Marcel du Toit, Zivanai C. Chapanduka, Annalise E. Zemlin

**Affiliations:** 1 Division of Haematological Pathology, Department of Pathology, National Health Laboratory Service, Tygerberg Academic Hospital, Faculty of Medicine and Health Sciences, Stellenbosch University, Cape Town, South Africa; 2 Division of Chemical Pathology, Department of Pathology, National Health Laboratory Service, Tygerberg Academic Hospital, Faculty of Medicine and Health Sciences, Stellenbosch University, Cape Town, South Africa; PLOS ONE, UNITED KINGDOM

## Abstract

**Background:**

Pre-analytical variables can have a significant adverse impact on the quality and credibility of coagulation test results. Therefore, correct and consistent identification of pre-analytical variables that compromise coagulation specimen quality is of paramount importance. Lack of standardization and heterogeneity among laboratory staff when assessing coagulation specimens can lead to inconsistent identification of these variables. Failure to recognize such pre-analytical variables results in the analysis of poor quality specimens and the authorization of spurious test results.

**Objectives:**

To determine the impact of a laboratory staff training workshop on coagulation specimen rejection rates and to ascertain the level of knowledge of laboratory personnel concerning coagulation specimen rejection criteria before and after the workshop.

**Methods:**

A retrospective three-month audit was performed with rejection data of incorrect blood to additive ratio, clotted, aged and haemolysed specimens collected. Training workshops and evaluation sessions were subsequently presented. A revised standard operating procedure delineating coagulation specimen rejection criteria was implemented and a repeat three-month audit was conducted.

**Results:**

In total, 13 162 coagulation specimens were received during the initial audit with 1 104 specimens (8.39%) rejected. Following the workshops, the rejection rate increased by 3.49% to 11.88% with 12 743 coagulation specimens received and 1 514 specimens rejected. Evaluation sessions performed before and after the workshops revealed that 95.2% of attendees attained improved knowledge.

**Conclusion:**

This study demonstrated the pivotal importance of regular laboratory staff training. The increase in specimen rejection following the workshops signifies their success in educating laboratory personnel regarding the correct identification of pre-analytical variables. Since most pre-analytical variables occur outside the laboratory, educational workshops need to be extended to non-laboratory personnel responsible for specimen collection and transport.

## Introduction

In clinical laboratory medicine, the process of specimen testing follows three sequential phases. These phases, the pre-analytical, analytical and post-analytical phase, constitute the total testing process [1]. The pre-analytical phase is defined by the International Organization for Standardization (ISO) 15189:2012 as “processes that start, in chronological order, from the clinician’s request and include the examination request, preparation and identification of the patient, collection of the primary sample(s), and transportation to and within the laboratory, and end when the analytical examination begins” [2, 3]. Research has revealed that although each of these three phases is susceptible to error, the pre-analytical phase is most vulnerable with nearly 70% of all laboratory errors occurring during this phase [4]. Further publications have documented an even greater prevalence of pre-analytical errors, with a one-year study by Goswami et al reporting that pre-analytical errors accounted for 77.1% of all laboratory errors [5]. This can be attributed to the comparatively high incidence of human errors that affect controllable variables during the pre-analytical phase [6]. In contrast, the incidence of errors during the analytical phase of testing has significantly declined in recent years mainly due to the improvements in analytical standardization and technological advancement in laboratory instrumentation [7]. Since pre-analytical variables are predominantly encountered outside the laboratory environment, errors affecting these variables frequently occur beyond the jurisdiction of laboratory personnel [8]. Research has illustrated that a deficiency of laboratory control during the pre-analytical phase is associated with a higher prevalence of poor quality specimens [9]. These studies revealed that sample collection by non-laboratory personnel accounted for the majority of rejected phlebotomy specimens [9, 10]. Insufficient knowledge and training of non-laboratory personnel with regard to the correct phlebotomy techniques and the subsequent deleterious effect of errors on the numerous quality sensitive pre-analytical variables is well known and commonly implicated [11]. The unfortunate reality of a deficiency in total laboratory quality control during the pre-analytical phase invariably necessitates laboratory staff to, both consistently and correctly identify specimens with pre-analytical errors that render them unsuitable for analysis. The significance is emphasized by studies yielding evidence that errors affecting controllable variables in the pre-analytical phase are known to have an adverse impact on the quality of blood samples, in particular coagulation specimens [12, 13]. These errors can lead to erroneous test results which expose the patient to unnecessary additional investigations and/or inaccurate diagnosis, inappropriate treatment and significant financial ramifications [14]. Quality assurance forms the cornerstone of every laboratory since the reputation and credibility of a laboratory is directly dependent on its ability to provide precise and accurate results. The importance of providing such high quality reliable results is emphasized by the fact that 60–70% of clinical decision making is based on laboratory results [14, 15].

In accordance with quality control processes, every laboratory should adhere to international laboratory standards. The ISO *15189 Medical laboratories–requirements for quality and competence*, which was launched in 2003, includes technical as well as management requirements for laboratories. The third edition, ISO 15189:2012, stipulates the quality management systems that laboratories should have in place in order to attain accreditation [2]. In South Africa, the government recognizes the South African National Accreditation System (SANAS) as the single body that can award competency for a laboratory to be recognized as accredited. According to SANAS, the quality policy of a laboratory states that the evaluation of laboratory performance is mandatory. The objective is to identify incorrect practices and deficiencies in knowledge, followed by the implementation of corrective procedures which is intrinsic to continuous laboratory quality improvement.

The importance of establishing concise quality control guidelines to aid laboratory personnel in the identification of errors affecting controllable pre-analytical variables is clearly evident [16]. This mandates the implementation of a standard operating procedure (SOP) that delineates precise specimen rejection criteria [17]. These guidelines require strict adherence by laboratory personnel to avoid heterogeneity and interpersonal bias. Poor adherence or absence of such protocols results in a loss of standardization among laboratory personnel and most likely increase laboratory error rate and negatively impact patient care by analysing poor quality specimens.

Previous studies identified the most common pre-analytical variables responsible for the rejection of phlebotomy specimens [18–20]. Studies in the field of haemostasis also recognized the most frequent pre-analytical variables implicated in coagulation specimen rejection [21]. Further literature concluded that among all laboratory specimens received, pre-analytical errors resulted in the highest rejection rates among coagulation specimens [22]. Collectively these studies illustrated that the quality of coagulation samples is sensitive to various technical and time variables. Deficiencies in the pre-analytical phase and exploring avenues of remedial action to reduce the incidence of pre-analytical errors have also been studied [23–27]. However, a critical analysis concerning the accuracy with which pre-analytical errors are identified in the haemostasis laboratory is lacking. Loss of harmonization among laboratory personnel with respect to the identification of pre-analytical errors can have serious implications and is contradictory to the quality control mandate of a laboratory. To our knowledge, no studies explored how accurately laboratory personnel assess pre-analytical errors nor the impact of laboratory staff training workshops on coagulation specimen rejection rates locally or internationally. The achieved objective of this study was to determine the impact of educational training workshops on coagulation specimen rejection rates and to ascertain the level of knowledge of laboratory personnel concerning coagulation specimen rejection criteria.

## Methods

### Ethical consideration

The study was granted ethical approval by the Health Research Ethics Committee (HREC), Stellenbosch University (ref. no. S17/09/175). In this absent patient contact study, a waiver of informed consent was approved. The study was performed in accordance with ethical guidelines as per the Declaration of Helsinki (2013).

### Study design

This study, consisting of four components, was conducted according to the principles of a quasi-experimental research design. Part one was an initial, pre-intervention retrospective audit where data of coagulation specimens received and rejected over a three-month period between 01 January 2018 to 31 March 2018 was collected. The four principal pre-analytical variables implicated in coagulation specimen rejection were identified and their respective rejection rates were determined. The second part of the study entailed the design and presentation of a structured theoretical and practical training workshop endorsed by Stellenbosch University with Continuous Professional Development (CPD) accreditation. The training workshop content consisted of (S1 Appendix):

- An overview of the importance of laboratory based audits
- The initial audit results of specimen rejection in the coagulation laboratory
- Ethical considerations in coagulation specimen processing
- Pre-analytical variables and coagulation specimen rejection criteria according to CLSI guidelines
- Principles, interpretation and result analysis of the Sysmex CS-2100i coagulation analyser
- Practical session in the laboratory with consolidation of theoretical knowledge

The target audience for the training workshop was the haematology laboratory personnel, focussing on haematological pathology residents, haematology technologists and student technologists. The training workshop was presented on three separate occasions (03 October 2018, 10 October 2018 and 17 October 2018) to accommodate all personnel. Course booklets containing training workshop lecture content was supplied to all participants. During training, the adherence to coagulation specimen rejection criteria as stipulated in the Clinical Laboratory Standards Institute (CLSI): *Collection*, *Transport*, *and Processing of Blood Specimens for Testing Plasma-Based Coagulation Assays and Molecular Haemostasis Assays; Approved Guideline–Fifth Edition*. *H21-A5* (ISBN 1-56238-657-3) was discussed and emphasized [28]. To assess knowledge of pre-analytical variables and criteria for coagulation specimen rejection, two critical written evaluation sessions were conducted on the same day of training, one immediately before and after the workshop. A two part questionnaire consisting of a knowledge assessment section and a practice assessment section was provided to each participant before the start of the training workshop. The knowledge assessment section contained a set of questions that explored the participants understanding of pre-analytical variables and rejection guidelines for coagulation specimens. A combination of questions requiring either single answers or more detailed explanations were included in the knowledge assessment section. The practice assessment section consisted of questions that investigated current practices within the coagulation laboratory (S2 Appendix). The questionnaire provided to participants after the conclusion of the training workshop comprised only of a knowledge assessment section where the same set of questions contained in the initial questionnaire was presented to participants. Questionnaires were completely anonymized. To enable comparison of a participants’ questionnaire before and after the training workshop a number was randomly assigned to a participant and indicated on the questionnaire. Three different questionnaires with non-identical knowledge assessment sections were prepared; one for each of the three training workshops (S3–S8 Appendices). The third part of the study was the implementation of a revised SOP for the coagulation laboratory with complete coagulation specimen rejection guidelines in accordance with the criteria contained in the CLSI H21-A5 document [28]. The SOP was revised in October 2018 after conclusion of the training workshop and made available to laboratory personnel immediately prior to the start of the second audit (S9 Appendix). The previous SOP (S10 Appendix) detailing coagulation specimen rejection criteria was subsequently replaced by the new amended SOP (S11 Appendix) on the National Health Laboratory Service (NHLS) electronic Q-pulse system. Emphasis was placed on a transparent guideline to avoid ambiguity and to allow effortless and continuous referencing. The fourth and final part of the study was a retrospective audit following the educational training workshops and the implementation of the new amended SOP containing the revised coagulation specimen rejection guidelines. The post-intervention audit commenced on 01 November 2018, four weeks after the first training workshop and two weeks after the third and final workshop. The number of coagulation specimens received over a three-month period between 01 November 2018 and 31 January 2019 was obtained and the specimen rejection rate resulting from the four principal pre-analytical variables was calculated.

The criteria according to which pre-analytical variables are assessed and how affected coagulation samples are handled is contained in the CLSI H21-A5 guidelines:

Incorrect blood to anticoagulant ratio: When considering optimal fill volumes the CLSI H21-A5 guidelines state that coagulation specimen collection tubes should be filled to a blood:anticoagulant ratio of 9:1. This requires blood collection to the manufacturers indicated optimal draw volume. Specimens within 10% of the optimal fill volume are considered acceptable for testing whilst quantity insufficient (< 90%) and overfilled coagulation specimens should be rejected. This guideline was implemented at our laboratory since no in-house studies were available to assess the impact of varying degrees of tube filling on test results. Reference coagulation tubes containing saline with 90% and 110% optimal fill volumes were placed in the laboratory to assist personnel and allow comparison with samples for analysis.

Aged specimens: The CLSI H21-A5 document states that laboratory staff should always assess specimen collection times prior to authorizing results. Clinicians who submit coagulation specimens to the laboratory for analysis are required to indicate the date and time of specimen collection on the request form. During the analytical phase of the total testing process the coagulation analyser will electronically record the time of specimen analysis. The time interval between specimen collection and analysis will be available to laboratory personnel and needs to be carefully reviewed prior to result authorization. This will ensure that values generated by aged specimens are not released to treating clinicians. When considering time to specimen analysis the acceptable interval from collection to testing depends on the type of coagulation assay performed. Activated partial thromboplastin time (aPTT) assays required for the monitoring of unfractionated heparin therapy should be centrifuged within one hour of collection. aPPT assays for non-heparinized patients should be analysed within four hours of specimen collection whilst a time delay of 24 hours is acceptable for prothrombin assays. Most other assays (thrombin time, protein C, factor V) require coagulation specimens to be centrifuged and analysed within four hours of collection. These time frames were emphasized during training workshops and incorporated in the revised SOP for the haemostasis laboratory.

Clotted specimens: Coagulation specimens containing blood clots are associated with inaccurate results and must be rejected. The CLSI H21-A5 guidelines indicate that all coagulation specimens should be evaluated for the presence of blood clots. This can be achieved by gentle inversion and observation or by carefully inserting and removing two wooden applicator sticks. Deviations from the recommended guidelines were identified in our laboratory and corrective measures were instituted.

Haemolysed specimens: Haemolysis is associated with the release of intracellular and membrane constituents that may result in clotting factor activation and inaccurate results. Haemolysis also changes the light transmittance properties of plasma and interferes with end-point clot detection when using an optical analyser. Our coagulation unit uses the Sysmex CS-2100i analyser with its photo-optical end-point clot detection method to generate coagulation test results. To identify the presence of an interfering substance, the analyser utilizes a multi-wavelength detection method and HIL (haemolysis, icterus and lipaemic) detector. The minimum detection concentration for haemolysis is a plasma haemoglobin level of approximately 40mg/dL. When this threshold is exceeded a “haemolytic sample error” message will be displayed as “Hem” on the job list of the Information Processing Unit (IPU) screen. The Sysmex CS-2100i analyser will flag the presence of haemolysis from level 0 to level 5 depending on the range of light absorbance and the concentration of haemoglobin in the plasma. Sysmex recommends level 1 (haemoglobin concentration of approximately 40mg/dL or greater) as the threshold level for haemolysis detection. The presence of a “Slight Coagulation”, “Analysis Time Over”, “Coagulation Curve Error” and “No Coagulation” error message should also prompt laboratory personnel to interrogate sample quality and investigate for the presence of haemolysis. Importantly, HIL analysis cannot be performed in micro mode for paediatric samples and laboratory personnel are required to identify and reject haemolysed paediatric samples based on visual assessment. Coagulation specimens from adult patients with evidence of gross haemolysis can reliably be rejected upon visual assessment. According to the CLSI H21-A5 guidelines haemolytic samples are generally considered incompatible for testing when using a photo-optical end-point detection analyser and as a result all haemolysed specimens noted on visual assessment or haemolytic samples identified by the analyser were rejected.

### Study site

The study was conducted at the National Health Laboratory Service (NHLS) Haematology Laboratory in Tygerberg Academic Hospital (TAH). TAH is a 1 384 bed multidisciplinary tertiary hospital located in Cape Town, South Africa that provides medical services to the public health sector. TAH is affiliated to Stellenbosch University and is the principal training hospital for undergraduate and postgraduate health care workers. The coagulation unit at the haematology laboratory provides a pathology service to TAH and also renders services to tertiary hospitals, district hospitals and regional clinics. The coagulation laboratory at TAH receives approximately 4000 to 4500 coagulation specimens per month.

### Study population

This study was confined to coagulation specimens and had no predetermined sample limit.

### Inclusion and exclusion criteria

All coagulation specimens received and analysed at the TAH coagulation laboratory during the research period were included in the study. Specimens collected at TAH but referred and analysed at a distant laboratory were excluded from the study since training workshops were only presented to TAH laboratory personnel.

### Data and statistical analysis

Data was captured using Microsoft Excel® software and Stata 15.1 statistical software was used for data analysis. Statistical results were generated with the assistance of a biostatistician affiliated to the Division of Epidemiology and Biostatistics at Stellenbosch University. The two sample test of proportions was used to compare coagulation specimen rejection and participants level of knowledge outcomes after training.

## Results

The four most prevalent pre-analytical variables identified in the study were incorrect coagulation tube fill volumes (i.e. incorrect blood to additive ratio), aged, clotted and haemolysed specimens. During the initial three-month audit from 01 January 2018 to 31 March 2018 a total of 13 162 specimens were received for processing at the TAH NHLS coagulation laboratory. Incorrect blood to additive ratios resulted in the rejection of 414 specimens which amounted to a 3.15% rejection rate of all specimens received. During the same period, 271 haemolysed specimens were identified and discarded, translating into a rejection rate of 2.06%. Clotted specimens constituted the third most prevalent pre-analytical variable implicated in specimen rejection with 246 samples rejected at a rate of 1.87%. Finally, 173 aged specimens were identified as unsuitable for analysis culminating in a rejection rate of 1.31%. Collectively, these four pre-analytical variables were responsible for the rejection of 1 104 coagulation specimens resulting in a rejection rate of 8.39% of all coagulation specimens received. Notably, these four pre-analytical variables accounted for 73.99% of all coagulation samples rejected.

**Fig 1:**
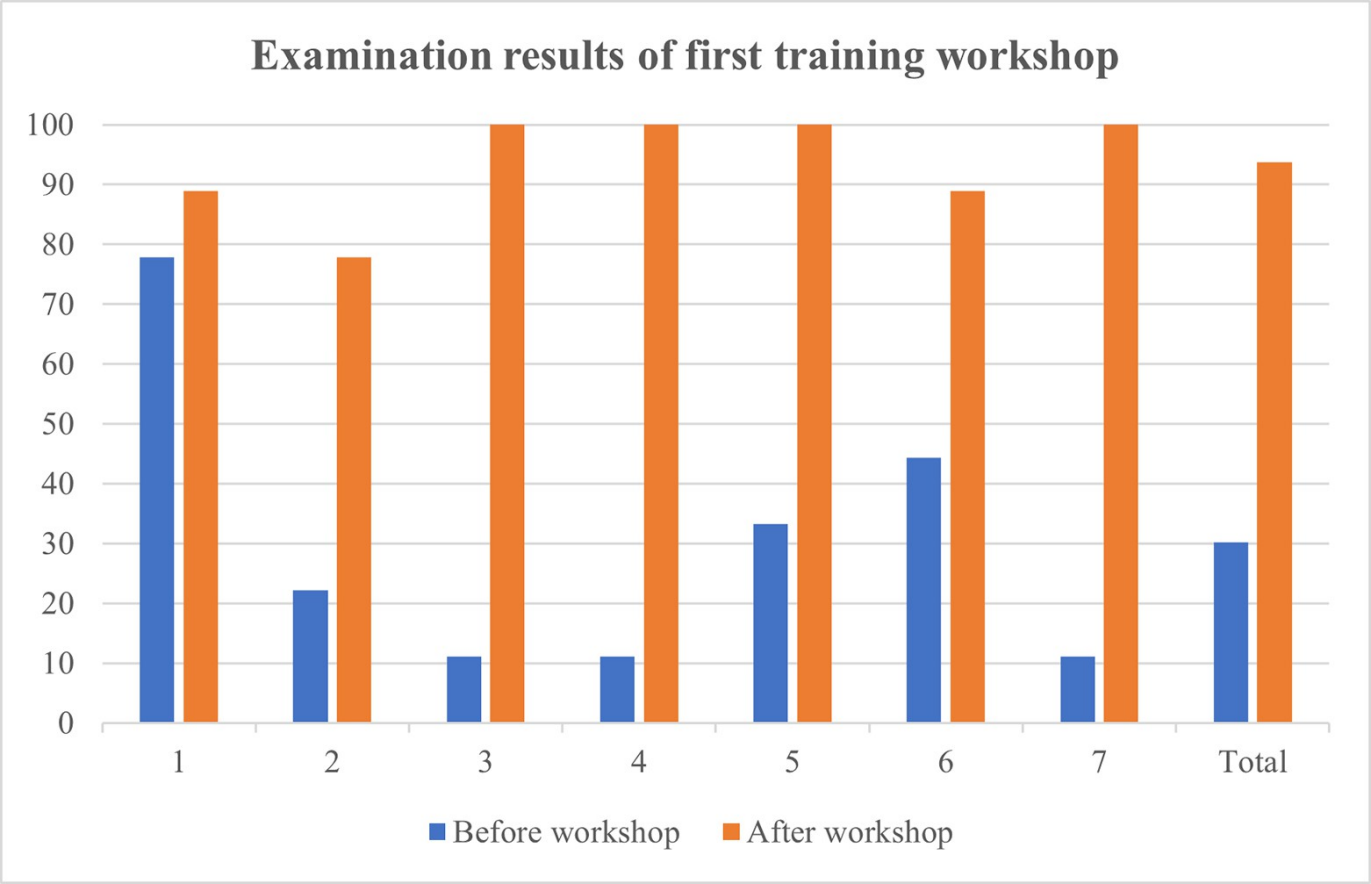
Comparison of participants’ knowledge assessment percentage results before and after the presentation of the first training workshop. 1 –participant 1; 2 –participant 2; 3 –participant 3; 4 –participant 4; 5 –participant 5; 6 –participant 6; 7 –participant 7.

**Fig 2:**
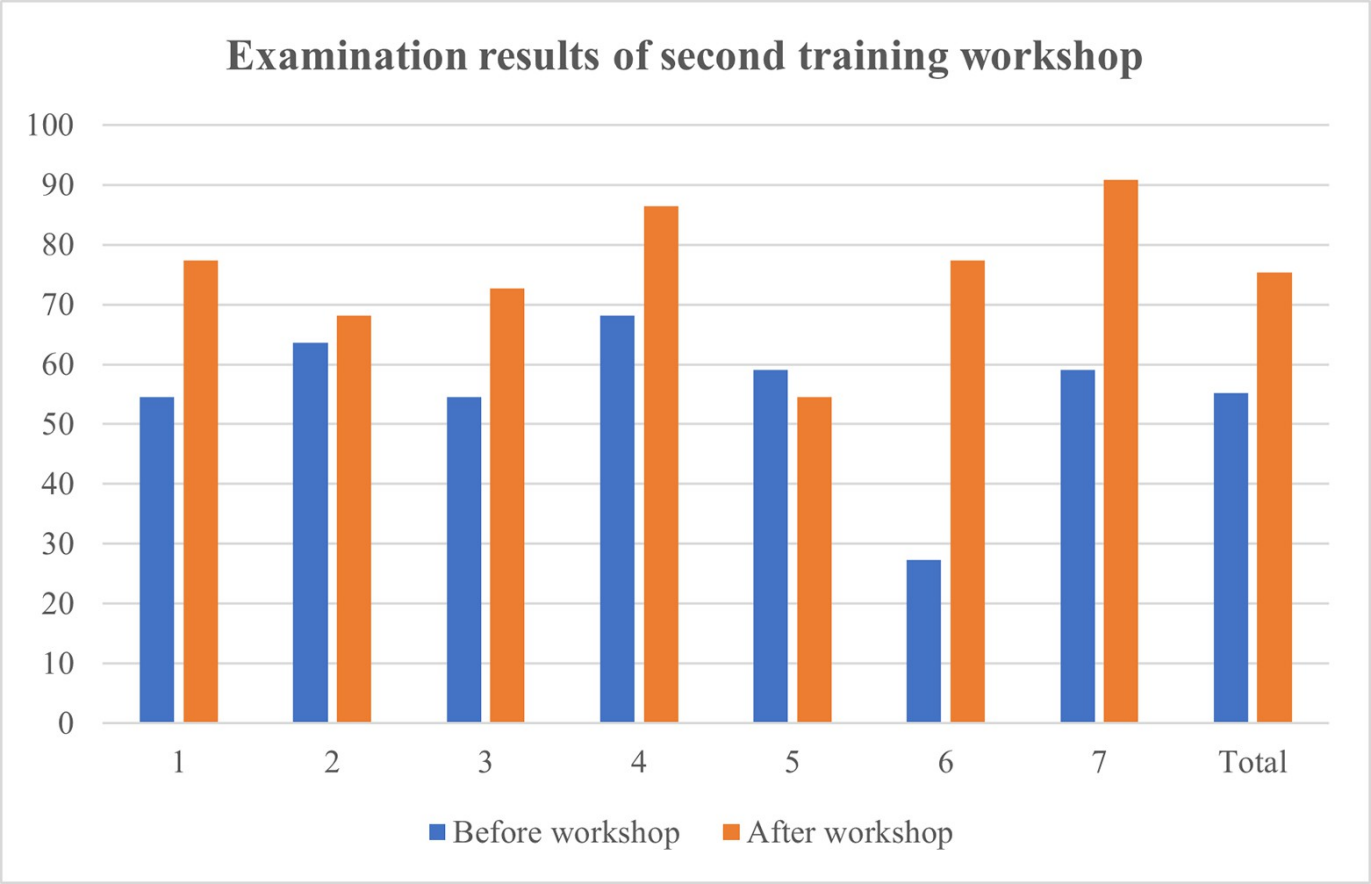
Comparison of participants’ knowledge assessment percentage results before and after the presentation of the second training workshop. 1 –participant 1; 2 –participant 2; 3 –participant 3; 4 –participant 4; 5 –participant 5; 6 –participant 6; 7 –participant 7.

**Fig 3:**
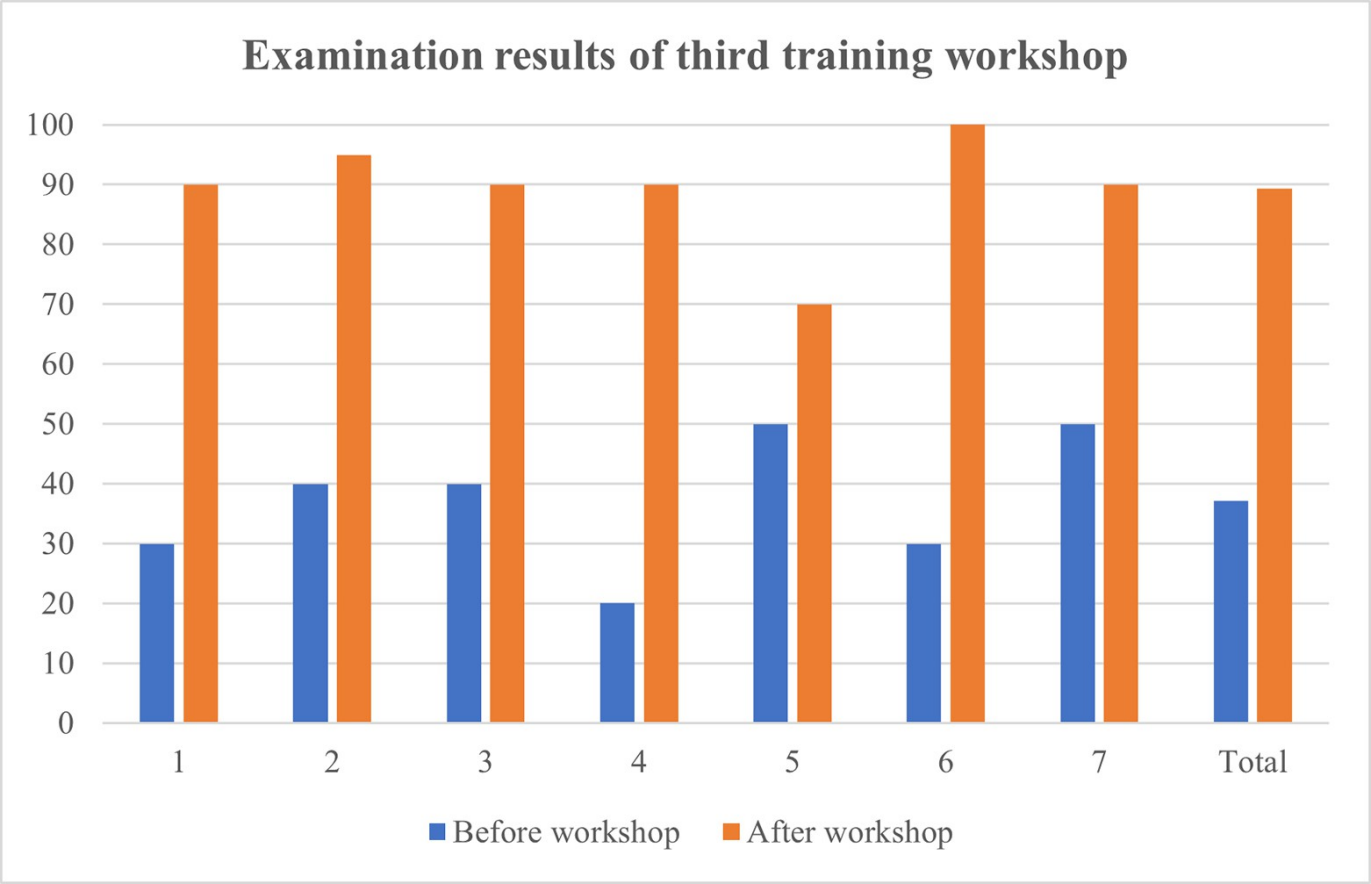
Comparison of participants’ knowledge assessment percentage results before and after the presentation of the third training workshop. 1 –participant 1; 2 –participant 2; 3 –participant 3; 4 –participant 4; 5 –participant 5; 6 –participant 6; 7 –participant 7.

**Fig 4:**
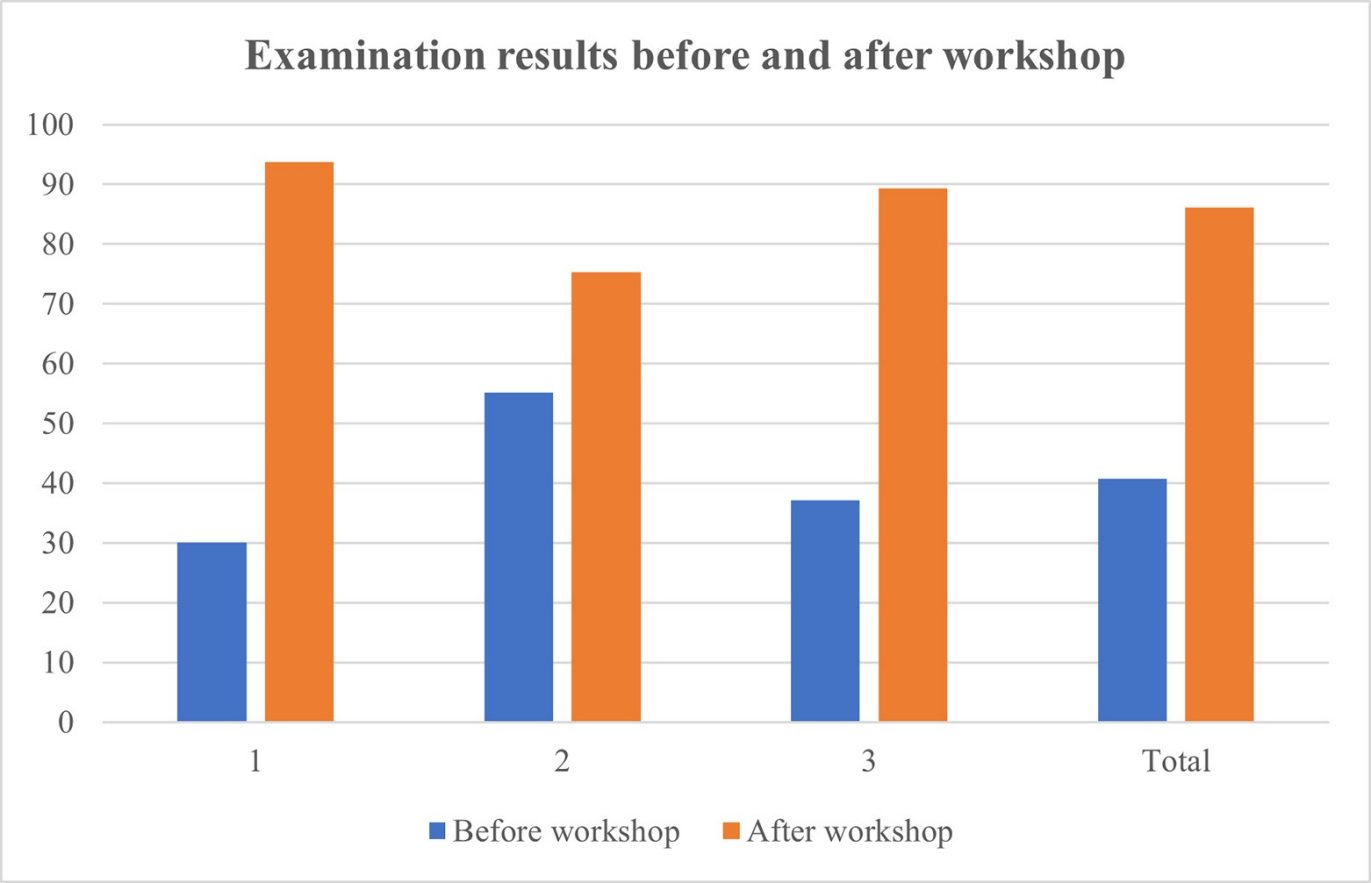
Comparison of combined knowledge assessment percentage results before and after training for all three workshops. 1 –first training workshop; 2 –second training workshop; 3 –third training workshop.

Training workshops were hosted on three separate days and managed to secure an attendance of 87.5% of all haematology laboratory personnel. A total of 21 haematology staff members consisting of haematological pathology residents, haematology technologists and student technologists participated in the training workshop. Written assessments before and after the workshop revealed that 95.2% of participating staff members achieved an overall improvement in knowledge. A significant improvement in results were obtained for each of the three training workshops following intervention (Table 1). The mean result achieved in the post-training knowledge assessment questionnaire increased by 105.1% when compared to the initial assessment. A practice assessment questionnaire completed by 20 haematology staff members confirmed the existence of heterogeneous and incorrect practices within the laboratory. As much as 75% (n = 15) of the attending staff members indicated that they do not consistently assess for the presence of a blood clot in all coagulation specimens. Only 45% (n = 9) of staff members provided a satisfactory explanation concerning the correct criteria and method of specimen fill volume assessment. Furthermore, 35% (n = 7) of staff members indicated that they do not always assess the specimen collection time prior to authorizing a result. Figs 1–4 illustrate the results attained before and after the educational measure for each of the three training workshops.

**Table 1:** Comparison of knowledge assessment results before and after training workshop intervention.

	Before training (%)	After training (%)	p-value	95% CI	
				Lower	Upper
Workshop 1	30.2	93.7			
Workshop 2	55.2	75.3			
Workshop 3	37.1	89.3			
Total	41.7	85.5	< 0.001	-0.520	-0.356

CI–Confidence Interval.

**Fig 5:**
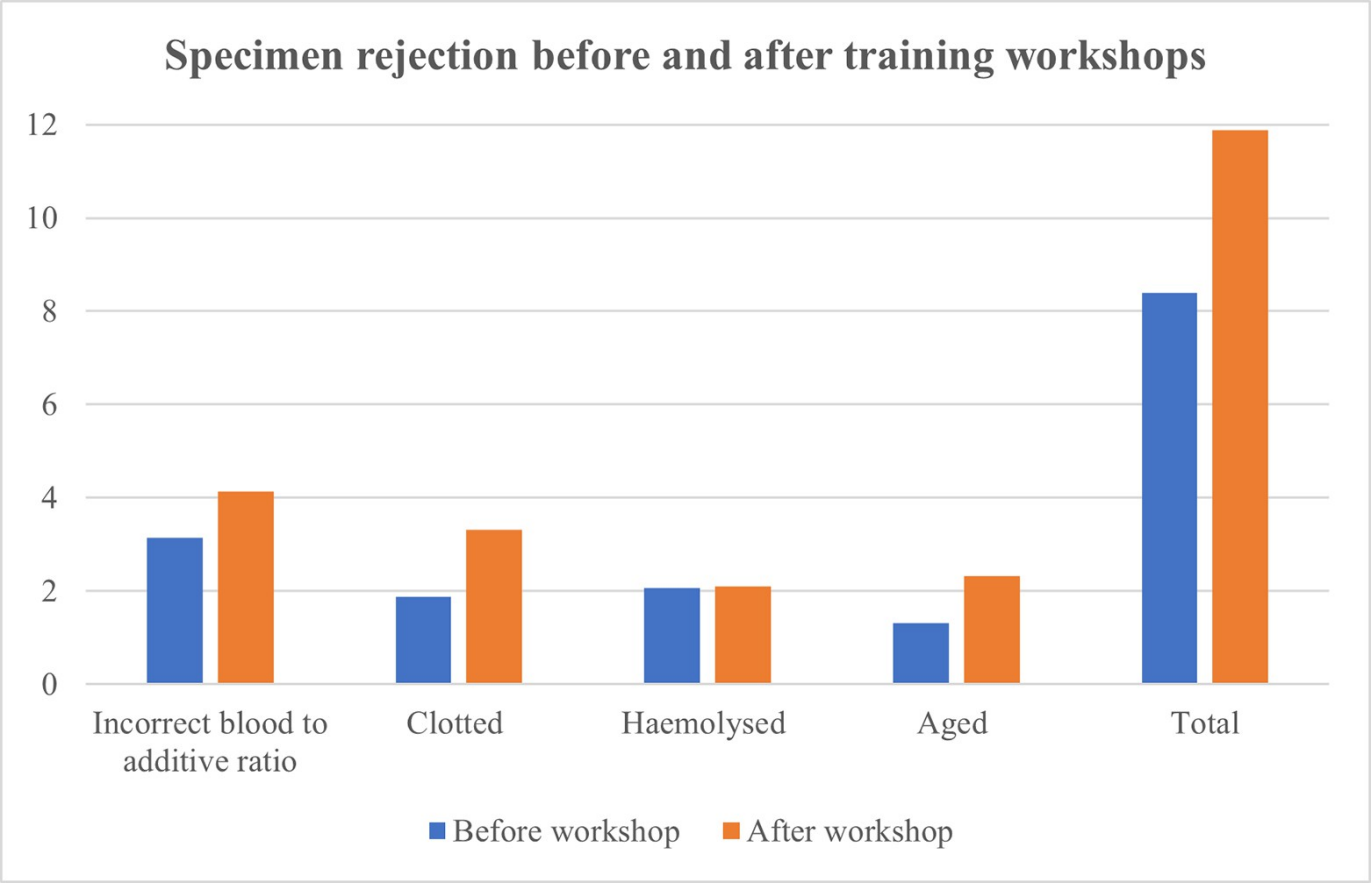
Comparison of coagulation specimen rejection percentages for each of the four principal pre-analytical variables before and after the presentation of training workshops.

Data collected during the second, post-intervention, three-month audit from 01 November 2018 to 31 January 2019 revealed that 12 743 samples were registered at the coagulation laboratory. Incorrect blood to additive ratios were responsible for the rejection of 527 coagulation specimens or 4.14% of all samples received. The second most prevalent pre-analytical variable resulting in specimen rejection was clotted samples. A total of 422 clotted coagulation specimens were discarded yielding a rejection rate of 3.31%. Aged specimens were responsible for the rejection of 297 samples or 2.33% of all coagulation specimens received. Finally, 268 haemolysed specimens were identified, accounting for a rejection rate of 2.10% of all registered coagulation specimens. Altogether, these four pre-analytical variables were responsible for the rejection of 1 514 specimens representing an 11.88% rejection rate of all coagulation specimens received. A comparison of coagulation specimen rejection before and after intervention is presented in Table 2 and Fig 5.

**Table 2:** Comparison of coagulation specimen rejection before and after training workshop intervention for each of the four principal pre-analytical variables.

Pre-analytical variable	First audit Pre-intervention (%)	Second audit Post-intervention (%)	p-value	95% CI	
				Lower	Upper
Incorrect blood to additive ratio	3.15	4.14	< 0.001	-0.014	-0.005
Haemolysed	2.06	2.10	0.822	-0.003	0.003
Clotted	1.87	3.31	< 0.001	-0.018	-0.010
Aged	1.31	2.33	< 0.001	-0.013	-0.006
Total	8.39	11.88	< 0.001	-0.042	-0.027

CI–Confidence Interval.

Finally, course evaluation forms revealed that 100% of all participants agreed that the training workshop was beneficial and added knowledge that can be practically applied in the haematology laboratory.

## Discussion

The objective of this study was to determine the impact of laboratory staff training workshops on coagulation specimen rejection and to ascertain the level of knowledge of laboratory personnel concerning current coagulation specimen rejection guidelines. The study focussed on the four principal pre-analytical variables implicated in specimen rejection at the TAH coagulation laboratory. A pre- and post-intervention audit determined an improvement in the detection of pre-analytical errors associated with poor quality coagulation specimens. This study was prompted by the existence of heterogeneity among laboratory personnel regarding the identification of pre-analytical variables that adversely affect coagulation specimen quality. The paucity of national and international published articles regarding the accuracy with which pre-analytical variables are identified by haemostasis laboratory personnel was further motivation for the study.

An integral component of laboratory medicine is clinical governance and can be defined as “a system through which national health service organizations are accountable for continually improving the quality of their services and safeguarding high standards of care by creating an environment in which excellence in clinical care will flourish” [29]. One of the pivotal components of clinical governance are audits that evaluate and compare current practices against critical evidence based guidelines. Audits can be described as “a quality improvement process that seeks to improve the patient care and outcomes through systematic review of care against explicit criteria and the implementation of change” [30]. Therefore, audits are instrumental in quality assurance by identifying deficiencies in current practice, implementing corrective measures and evaluating the impact of such intervention to ultimately provide the highest standard of service and patient care.

Quality indicators of coagulation specimens are specified by the CLSI, an international, standards developing and educational organization that promotes the use and development of consensus standards and guidelines within the healthcare community [28]. The CLSI H21-A5: *Collection*, *Transport*, *and Processing of Blood Specimens for Testing Plasma-Based Coagulation Assays and Molecular Haemostasis Assays; Approved Guideline–Fifth Edition* provides distinct guidelines concerning the preparation and handling of coagulation specimens. It describes the quality requirements of numerous pre-analytical variables and enables the critical appraisal of coagulation specimen integrity. The guideline was developed to aid laboratory personnel who are responsible for evaluating coagulation specimen quality prior to instrument analysis. CLSI H21-A5 endeavours to establish uniformity in coagulation specimen preparation and handling and promotes the identification of variables that negatively impact on coagulation test results. Pre-analytical variables outlined in CLSI H21-A5 that demand laboratory staff scrutiny include specimen labelling, completion of test request forms, specimen collection tubes containing the correct type and percentage concentration of anticoagulant, specimen transport, specimen collection tube fill volume, time to specimen analysis and identifying the presence of blood clots, haemolysis, elevated haematocrit, icteric and lipaemic specimens.

Despite numerous pre-analytical variables that compromise coagulation specimen quality, the four principal variables implicated in sample rejection during our two audits were incorrect blood to additive ratio, clotted, haemolysed and aged specimens. Following the training workshops, an increase in rejection amongst all four pre-analytical variables was observed. The rejection rate varied from a 0.04% increase in haemolysed specimens to a 1.44% increase in rejection of clotted samples. The difference in rejection of haemolysed specimens between the initial and post-intervention audit was statistically insignificant. This can likely be attributed to the coagulation analysers high level of sensitivity and accuracy in detecting the presence of haemolysis. The reliance on laboratory personnel to identify this pre-analytical variable with the possibility of accompanying human error is subsequently reduced. A statistically significant increase in rejection was noted among incorrect blood to additive ratio and aged specimens. Rejection of coagulation specimens due to the presence of a blood clot also increased during the second audit period and represents the pre-analytical variable responsible for the greatest post-intervention increase in coagulation specimen rejection. When considering these four pre-analytical variables, an overall increase in specimen rejection was observed in the second audit.

Results obtained from the knowledge assessment questionnaire revealed that there was a statistically significant improvement in knowledge following the educational measure. Post-intervention written assessments demonstrated a marked improvement in results when compared to the results achieved during the initial assessment for all three workshops. The combined mark for the three training workshops showed a statistically significant increase after the training. Despite hosting the training workshop on three separate days, not all haematology laboratory personnel were able to attend. After the training workshops concluded and before the repeat audit commenced a booklet containing course material presented during the training workshop was placed in the coagulation laboratory. Personnel who were unable to attend the training workshops therefore still had access to the course content in the booklet.

Although the haemostasis laboratory at TAH had a SOP detailing the quality requirements of coagulation specimens, we found that not all pre-analytical variables associated with poor quality specimens were listed (S10 Appendix). The SOP was archived and proved difficult to locate thereby precluding effortless referencing by laboratory personnel. Following the training workshops an amended SOP was established setting forth comprehensive and current quality indicators and rejection guidelines (S11 Appendix). The new SOP was strategically placed in the coagulation unit to make it readily accessible to laboratory personnel.

To our knowledge, no previous studies explored the impact of laboratory staff training workshops on coagulation specimen rejection. Furthermore, the accuracy with which pre-analytical variables are identified by personnel working in the coagulation laboratory was not previously examined. Training workshops offered in past studies focussed primarily on non-laboratory personnel involved in specimen collection, handling and transport during the pre-analytical phase [24–26]. These studies identified the impact of such training by determining sample rejection rates before and after intervention. A recent quasi-experimental study performed at TAH revealed that physician education failed to improve the quality and comprehensiveness of thrombophilia screening test request forms [27]. A new, amended request form, electronic test ordering and continued physician education was proposed as solution.

Our study identified the presence of heterogeneous practices and lack of standardization within the laboratory as reflected in the practice assessment questionnaire. The importance of regular staff training was supported by the significant improvement in knowledge among laboratory personnel following the workshop. Educational programmes and continuous professional development workshops are however not always readily available for laboratory personnel. The consequences are that staff may not be adequately informed about new or revised rejection criteria for inadequate coagulation samples.

### Strengths and limitations of the study

A strength of this study was firstly its representative nature with both audits containing a large number of specimens. A further strength was the written assessments that demonstrated an improvement in knowledge among laboratory personnel. Limitations of the study included the inability to secure complete staff participation with only 87.5% of coagulation laboratory personnel attending the training workshop. A further limitation to the study was the inability to determine whether coagulation specimens received from referral facilities for aPTT analysis in patients on unfractionated heparin infusion therapy was centrifuged within one hour of collection. Another limitation was the exclusion of specialized coagulation tests not conducted at TAH and referred to distant laboratories for analysis. Finally, the workshop was confined to laboratory personnel and not healthcare workers involved in blood collection where most pre-analytical errors occur.

## Conclusion

This novel study unequivocally emphasizes the importance of regular laboratory staff training and the necessity of comprehensive and current international standardized guidelines within the laboratory. This will effectively reduce the number of poor quality coagulation specimens analysed and ensure the release of credible results that are imperative to patient care and safety. Since a large proportion of pre-analytical variables occur outside the laboratory environment, extending training workshops to healthcare workers responsible for specimen collection should be considered mandatory. Assessing the impact of such training sessions on coagulation specimen rejection rates is an area for future research.

## Supporting information

S1 Data (XLSX)

S2 Data (XLSX)

S3 Data (XLSX)

S4 Data (XLSX)

S5 Data (XLSX)

S6 Data (XLSX)

S7 Data (XLSX)

S8 Data (XLSX)

S9 Data (XLSX)

S10 Data (XLSX)

S1 Appendix (PDF)

S2 Appendix (PDF)

S3 Appendix (PDF)

S4 Appendix (PDF)

S5 Appendix (PDF)

S6 Appendix (PDF)

S7 Appendix (PDF)

S8 Appendix (PDF)

S9 Appendix (PDF)

S10 Appendix (PDF)

S11 Appendix (PDF)
