# Supplementary material for: The impact of laboratory staff training workshops on coagulation specimen rejection rates
Source: PLoS One. 2022 Jun 3;17(6):e0268764. doi: 10.1371/journal.pone.0268764 (PMC9165799; doi:10.1371/journal.pone.0268764)
Supplement: S1 Appendix — (PDF) [file pone.0268764.s011.pdf]

COURSE CONTENT

Time	Activity
11:30 – 11:40	Welcoming and introduction
11:40 – 11:50	Knowledge and practice assessment questionnaire
11:50 – 12:05	An overview of the importance of laboratory based audits
12:05 – 12:20	Specimen rejection in the Coagulation Laboratory (TAH): Initial audit results
12:20 – 13:20	Ethical considerations in coagulation specimen processing
13:20 – 14:00	Pre-analytical variables and specimen rejection according to CLSI H21-A5 guidelines
14:00 – 14:20	BREAK
14:20 – 15:00	Sysmex CS-2100i coagulation analyser – Principals, interpretation and result analysis
15:00 – 15:45	Practical session in laboratory
15:45 – 16:00	Knowledge assessment questionnaire
