# Supplementary material for: The impact of laboratory staff training workshops on coagulation specimen rejection rates
Source: PLoS One. 2022 Jun 3;17(6):e0268764. doi: 10.1371/journal.pone.0268764 (PMC9165799; doi:10.1371/journal.pone.0268764)
Supplement: S2 Appendix — (PDF) [file pone.0268764.s012.pdf]

Participant number:

Registrar ☐

Technologist ☐

QUESTIONNAIRE

1) Do you always assess the specimen collection time prior to authorizing a coagulation test result?

Yes	☐
No	☐

2) What is your action if no collection time is stated on the request form?

   a. Reject the result…………………………………………………………………… ☐
   b. Authorize the result……………………………………………………………….. ☐
   c. Authorize with a comment to treat the result with reserve….. ☐

3) Do you assess **ALL** coagulation specimens for evidence of a blood clot?

Yes	☐
No	☐

4) How do you assess if a specimen is within the correct fill volume?

____

____

5) Do you always inspect a coagulation specimen and look for an elevated hematocrit?

Yes	☐
No	☐

6) For icteric specimens, are you:

a. Rejecting the result………………………………………………………………… ☐
b. Authorizing the result…………………………………………………………… ☐
c. Authorizing with a comment to treat the result with reserve.. ☐
