# Supplementary material for: The impact of laboratory staff training workshops on coagulation specimen rejection rates
Source: PLoS One. 2022 Jun 3;17(6):e0268764. doi: 10.1371/journal.pone.0268764 (PMC9165799; doi:10.1371/journal.pone.0268764)
Supplement: S3 Appendix — (PDF) [file pone.0268764.s013.pdf]

Participant number:

Registrar ☐

Technologist ☐

QUESTIONNAIRE

03 OCTOBER 2018

1) What is the function of the sodium citrate additive in coagulation specimen collection tubes? (1 mark)

____

____

2) What are the two different percentage concentrations of sodium citrate used in coagulation specimen collection tubes? (2 marks)

____

____

3) What is the effect of the higher concentration of sodium citrate additive on the clotting time of a specimen? (1 mark)

____

____

4) What is the correct blood to additive ratio for coagulation specimens? (1 mark)

____

____

5) How much percentage variation from the optimal fill volume is acceptable? (1 mark)

____

____

6) Above what hematocrit level must a coagulation specimen be rejected? (answer in percentage or L/L) (1 mark)

____

7) What is the effect of an elevated hematocrit on the clotting time of a specimen? (1 mark)

____

____

8) The HIL (Haemolysis; Icterus; Lipemia) check is done on all samples? - True/False (1 mark)

____
