# Supplementary material for: The impact of laboratory staff training workshops on coagulation specimen rejection rates
Source: PLoS One. 2022 Jun 3;17(6):e0268764. doi: 10.1371/journal.pone.0268764 (PMC9165799; doi:10.1371/journal.pone.0268764)
Supplement: S4 Appendix — (PDF) [file pone.0268764.s014.pdf]

Participant number:

Registrar ☐

Technologist ☐

QUESTIONNAIRE

03 OCTOBER 2018

1) **Q:** What is the function of the sodium citrate additive in coagulation specimen collection tubes? (1 mark)

   A: *Sodium citrate acts as an anticoagulant by chelating the calcium present in the blood specimen. This produces a calcium free blood sample and prevents blood clot formation before testing is commenced.*

2) **Q:** What are the two different percentage concentrations of sodium citrate used in coagulation specimen collection tubes? (2 marks)

   A: *3.2% and 3.8%*

3) **Q:** What is the effect of the higher concentration of sodium citrate additive on the clotting time of a specimen? (1 mark)

   A: *It results in prolongation of the clotting time.*

4) **Q:** What is the correct blood to additive ratio for coagulation specimens? (1 mark)

   A: *9:1*

5) **Q:** How much percentage variation from the optimal fill volume is acceptable? (1 mark)

   A: *10%*

6) **Q:** Above what hematocrit level must a coagulation specimen be rejected? (answer in percentage or L/L) (1 mark)

   A: *Above 55% (0.55L/L)*

7) **Q:** What is the effect of an elevated hematocrit on the clotting time of a specimen? (1 mark)

   A: *It results in prolongation of the clotting time.*

8) **Q:** The HIL (Haemolysis; Icterus; Lipemia) check is done on all samples? - True/False (1 mark)

   A: *False*
