# Supplementary material for: The impact of laboratory staff training workshops on coagulation specimen rejection rates
Source: PLoS One. 2022 Jun 3;17(6):e0268764. doi: 10.1371/journal.pone.0268764 (PMC9165799; doi:10.1371/journal.pone.0268764)
Supplement: S5 Appendix — (PDF) [file pone.0268764.s015.pdf]

Participant number:

Registrar ☐

Technologist ☐

QUESTIONNAIRE

10 OCTOBER 2018

1) What is the maximum allowable time interval between specimen collection and specimen testing for PT/INR? (1 mark)

____

____

2) What is the effect of an underfilled collection tube on the clotting time of a specimen? (1 mark)

____

____

3) Coagulation specimens with haematocrits below 20% require sodium citrate adjustments? True or False. (1 mark)

____

____

4) Results from hemolyzed coagulation specimens may be authorized when using an electro-mechanical end point detection analyser. True or False? (1 mark)

____

____

5) Results from lipemic & icteric coagulation specimens may be authorized when using an electro-mechanical end point detection analyser. True or False? (1 mark)

____

____

6) What is the maximum time delay between specimen collection and centrifugation of a coagulation specimen for aPTT testing when taken from a patient receiving unfractionated heparin (UFH)? (1 mark)

____

____

7) What should be done in cases where a patient has an elevated hematocrit to still ensure reliable coagulation results? (1 mark)

____

____

8) What procedure will you follow if the coagulation analyser flags an early reaction error? (2 marks)

____

____

____

____

9) Please review the graph in addendum A. Explain how you would proceed. (2 marks)

____

____

____

____

____

ADDENDUM A

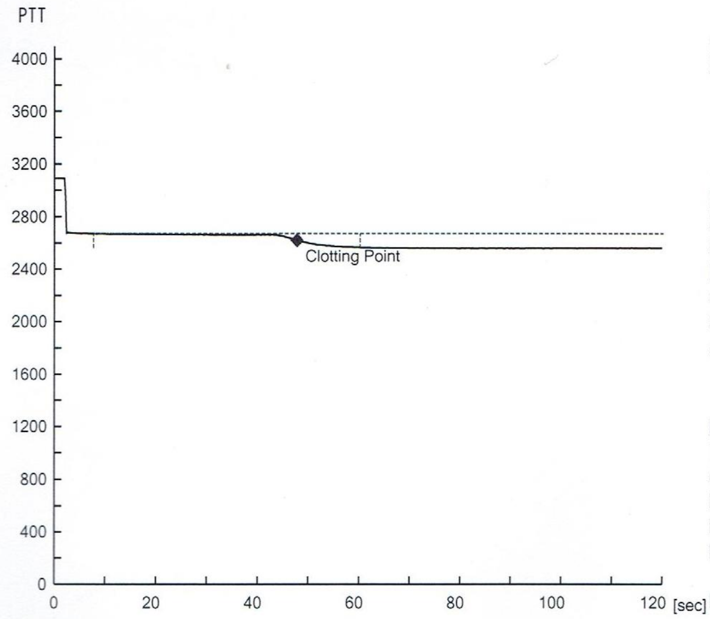

Result
PTT * 47.8 sec

Evaluation Info.

bH_point_time	7.7
bH	2672
End_point_time	60.2
dH	105
Coag.%	50
dOD	0.0174

Measurement Info.

Temperature	36.9
Channel No.	7
Management ID	5231
Dilution Ratio	1 / 1
Reagent Lot	APTT FS 538533
	CaCl2 563822
Elapsed Time	APTT FS 9
	CaCl2 8

Detail
0002.0000.0000 Slight Coagulation

Hem Detection Level
Ict Detection Level
Lip Detection Level
Vol Detection Level

Evaluation Data

1%	9.9	21%	45.1		41%	47.1	61%	49.1	81%	52.3
2%	15.4	22%	45.2		42%	47.2	62%	49.2	82%	52.5
3%	18.3	23%	45.3		43%	47.2	63%	49.3	83%	52.7
4%	19.6	24%	45.5		44%	47.4	64%	49.5	84%	52.8
5%	22.2	25%	45.5		45%	47.4	65%	49.6	85%	53.0
6%	24.6	26%	45.7		46%	47.5	66%	49.7	86%	53.2
7%	26.6	27%	45.8		47%	47.6	67%	49.8	87%	53.4
8%	30.4	28%	45.8		48%	47.6	68%	49.9	88%	53.8
9%	33.6	29%	45.9		49%	47.7	69%	50.1	89%	54.3
10%	43.7	30%	46.0	->	50%	47.8	70%	50.3	90%	54.7
11%	43.8	31%	46.1		51%	48.0	71%	50.4	91%	54.9
12%	43.9	32%	46.1		52%	48.0	72%	50.4	92%	55.1
13%	44.0	33%	46.3		53%	48.3	73%	50.5	93%	55.7
14%	44.3	34%	46.4		54%	48.4	74%	50.7	94%	56.1
15%	44.4	35%	46.5		55%	48.5	75%	50.9	95%	56.3
16%	44.6	36%	46.7		56%	48.6	76%	51.0	96%	56.6
17%	44.7	37%	46.7		57%	48.6	77%	51.1	97%	57.7
18%	44.8	38%	46.8		58%	48.7	78%	51.3	98%	58.6
19%	44.9	39%	46.8		59%	48.8	79%	51.4	99%	58.8
20%	45.0	40%	47.0		60%	48.9	80%	51.8	100%	60.3

12542/01-65 build 10 1/1 15/09/2018 03:50
