# Supplementary material for: The impact of laboratory staff training workshops on coagulation specimen rejection rates
Source: PLoS One. 2022 Jun 3;17(6):e0268764. doi: 10.1371/journal.pone.0268764 (PMC9165799; doi:10.1371/journal.pone.0268764)
Supplement: S6 Appendix — (PDF) [file pone.0268764.s016.pdf]

Participant number:

Registrar ☐

Technologist ☐

QUESTIONNAIRE

10 OCTOBER 2018

1) **Q:** What is the maximum allowable time interval between specimen collection and specimen testing for PT/INR? (1 mark)

   A: *24 hours*

2) **Q:** What is the effect of an underfilled collection tube on the clotting time of a specimen? (1 mark)

   A: *It results in prolongation of the clotting time.*

3) **Q:** Coagulation specimens with haematocrits below 20% require sodium citrate adjustments? True or False. (1 mark)

   A: *False*

4) **Q:** Results from hemolysed coagulation specimens may be authorized when using an electro-mechanical end point detection analyser. True or False? (1 mark)

   A: *False*

5) **Q:** Results from lipemic & icteric coagulation specimens may be authorized when using an electro-mechanical end point detection analyser. True or False? (1 mark)

   A: *True*

6) **Q:** What is the maximum time delay between specimen collection and centrifugation of a coagulation specimen for aPTT testing when taken from a patient receiving unfractionated heparin (UFH)? (1 mark)

   A: *1 hour*

7) **Q:** What should be done in cases where a patient has an elevated hematocrit to still ensure reliable coagulation results? (1 mark)

   A: *The coagulation specimen with an elevated haematocrit (>55%) results in falsely prolonged clotting times and therefore needs to be rejected. A modified coagulation collection tube has to be prepared where a specific quantity of sodium citrate is removed. The volume of sodium citrate that needs to be removed can be calculated from an equation or normogram chart. The treating clinician needs to be informed and the modified collection tube can be collected from the laboratory for blood sampling.*

8) **Q:** What procedure will you follow if the coagulation analyser flags an early reaction error? (2 marks)

   A: *Verify sample and reagent integrity. Assess for the presence of a blood clot. Visual inspection of the coagulation analyser reaction curve. Repeat measurement. Repeat blood sampling if error persists.*

9) **Q:** Please review the graph in addendum A. Explain how you would proceed (2 marks)

   A: *Verify sample integrity. Assess for the presence of haemolysis, lipemia and possible anticoagulant contamination. Verify delivery of sample and reagent. Review generated analyser data for clot formation (weak clot formation may be due to an abnormally low fibrinogen, factor deficiencies or the presence of inhibitors). Repeat sample measurement.*

ADDENDUM A

PTT

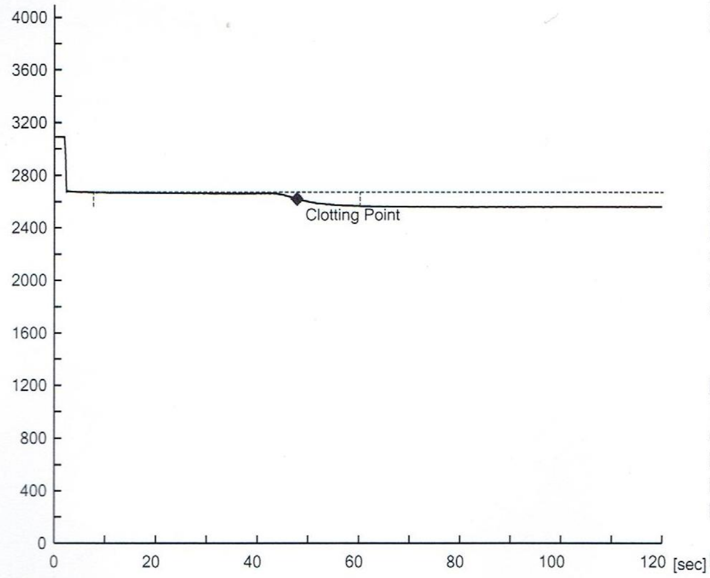

Detail
0002.0000.0000 Slight Coagulation

Result
PTT * 47.8 sec

Evaluation Info.

bH_point_time	7.7
bH	2672
End_point_time	60.2
dH	105
Coag.%	50
dOD	0.0174

Measurement Info.

Temperature	36.9	
Channel No.	7	
Management ID	5231	
Dilution Ratio	1 / 1	
Reagent Lot	APTT FS	538533
	CaCl2	563822
Elapsed Time	APTT FS	9
	CaCl2	8

Hem Detection Level
Ict Detection Level
Lip Detection Level
Vol Detection Level

Evaluation Data

1%	9.9	21%	45.1		41%	47.1	61%	49.1	81%	52.3
2%	15.4	22%	45.2		42%	47.2	62%	49.2	82%	52.5
3%	18.3	23%	45.3		43%	47.2	63%	49.3	83%	52.7
4%	19.6	24%	45.5		44%	47.4	64%	49.5	84%	52.8
5%	22.2	25%	45.5		45%	47.4	65%	49.6	85%	53.0
6%	24.6	26%	45.7		46%	47.5	66%	49.7	86%	53.2
7%	26.6	27%	45.8		47%	47.6	67%	49.8	87%	53.4
8%	30.4	28%	45.8		48%	47.6	68%	49.9	88%	53.8
9%	33.6	29%	45.9		49%	47.7	69%	50.1	89%	54.3
10%	43.7	30%	46.0	->	50%	47.8	70%	50.3	90%	54.7
11%	43.8	31%	46.1		51%	48.0	71%	50.4	91%	54.9
12%	43.9	32%	46.1		52%	48.0	72%	50.4	92%	55.1
13%	44.0	33%	46.3		53%	48.3	73%	50.5	93%	55.7
14%	44.3	34%	46.4		54%	48.4	74%	50.7	94%	56.1
15%	44.4	35%	46.5		55%	48.5	75%	50.9	95%	56.3
16%	44.6	36%	46.7		56%	48.6	76%	51.0	96%	56.6
17%	44.7	37%	46.7		57%	48.6	77%	51.1	97%	57.7
18%	44.8	38%	46.8		58%	48.7	78%	51.3	98%	58.6
19%	44.9	39%	46.8		59%	48.8	79%	51.4	99%	58.8
20%	45.0	40%	47.0		60%	48.9	80%	51.8	100%	60.3
