# Supplementary material for: The impact of laboratory staff training workshops on coagulation specimen rejection rates
Source: PLoS One. 2022 Jun 3;17(6):e0268764. doi: 10.1371/journal.pone.0268764 (PMC9165799; doi:10.1371/journal.pone.0268764)
Supplement: S7 Appendix — (PDF) [file pone.0268764.s017.pdf]

Participant number:

Registrar ☐

Technologist ☐

QUESTIONNAIRE

17 OCTOBER 2018

1) What is the maximum allowable time interval between specimen collection and testing for aPTT? (Please do not state a range) (1 mark)

____

____

2) Is the transportation of whole blood coagulation specimens on ice acceptable? Explain. (2 marks)

____

____

3) For coagulation collection tubes, what percentage fill/draw volume is considered underfilled? (1 mark)

____

____

4) For coagulation collection tubes, what percentage fill/draw volume is considered overfilled? (1 mark)

____

____

5) What procedure should be followed when receiving a specimen for coagulation assays in a patient with a haematocrit > 55%? (1 mark)

____

____

____

____

6) Optimal centrifugation is paramount to ensure platelet poor plasma. What should the platelet count be for a specimen to be considered platelet poor? (1 mark)

____

____

7) How often should the centrifugation procedure be validated? (1 mark)

____

____

8) Please review the graph in addendum A. Explain how you would proceed. (2 marks)

____

____

____

____

____

ADDENDUM A

CS-2100i NHLS Tygerberg admin : administrator

***** Graph Detail Print *****

Sample No:	ST02132505 B	Seq:	F
Rack Tube Pos:	ST0213-01	Re-analysis flag:	
Status:	Review	Validate:	Performed
S. Code:		Meas. Date:	06/09/2018 09:26:56
Analysis Mode:	Normal Cp		
Patient Name:	ROBERT		
Sample Comment:			
Sample Info.:	-		

PTT

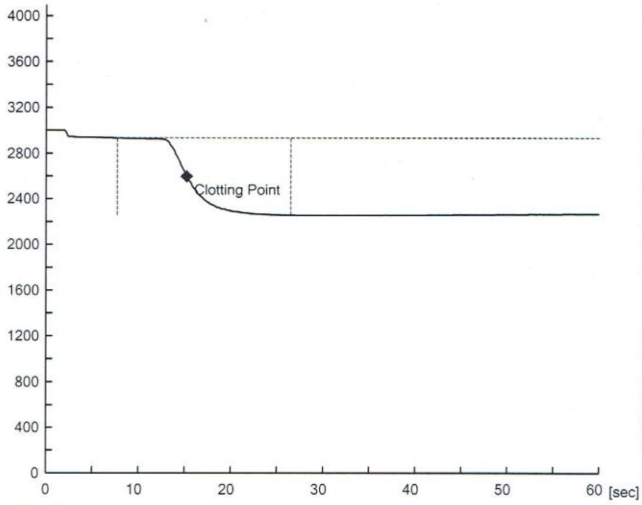

Result
PTT *****.* sec

Evaluation Info.
bH_point_time 7.7
bH 2935
End_point_time 26.5
dH 677
Coag.% 50
dOD 0.1139

Measurement Info.
Temperature 36.9
Channel No. 5
Management ID 5231
Dilution Ratio 1 / 1
Reagent Lot APTT FS 538533
CaCl2 563822
Elapsed Time APTT FS 23
CaCl2 7

Detail
0008.0128.0016 Early Reaction Error : Early %

Hem Detection Level 0
Ict Detection Level 0
Lip Detection Level 0
Vol Detection Level

Evaluation Data

1%	10.5	21%	14.0	41%	14.8	61%	15.7	81%	17.2
2%	12.7	22%	14.0	42%	14.8	62%	15.7	82%	17.3
3%	12.9	23%	14.1	43%	14.8	63%	15.8	83%	17.3
4%	13.0	24%	14.1	44%	14.9	64%	15.9	84%	17.4
5%	13.1	25%	14.1	45%	14.9	65%	15.9	85%	17.6
6%	13.2	26%	14.2	46%	15.0	66%	16.0	86%	17.8
7%	13.2	27%	14.2	47%	15.0	67%	16.0	87%	17.9
8%	13.3	28%	14.2	48%	15.1	68%	16.0	88%	18.0
9%	13.4	29%	14.2	49%	15.1	69%	16.1	89%	18.3
10%	13.4	30%	14.3	-> 50%	15.2	70%	16.2	90%	18.5
11%	13.5	31%	14.3	51%	15.2	71%	16.2	91%	18.6
12%	13.6	32%	14.4	52%	15.3	72%	16.3	92%	19.0
13%	13.6	33%	14.4	53%	15.3	73%	16.4	93%	19.3
14%	13.6	34%	14.5	54%	15.4	74%	16.5	94%	19.6
15%	13.6	35%	14.5	55%	15.4	75%	16.6	95%	20.1
16%	13.7	36%	14.6	56%	15.4	76%	16.7	96%	20.5
17%	13.7	37%	14.6	57%	15.4	77%	16.7	97%	21.2
18%	13.8	38%	14.7	58%	15.5	78%	16.8	98%	22.1
19%	13.9	39%	14.7	59%	15.5	79%	16.9	99%	23.5
20%	13.9	40%	14.8	60%	15.6	80%	17.0	100%	26.5

12542/01-65 build 10 1/1 15/09/2018 04:10
