# Supplementary material for: The impact of laboratory staff training workshops on coagulation specimen rejection rates
Source: PLoS One. 2022 Jun 3;17(6):e0268764. doi: 10.1371/journal.pone.0268764 (PMC9165799; doi:10.1371/journal.pone.0268764)
Supplement: S8 Appendix — (PDF) [file pone.0268764.s018.pdf]

Participant number:

Registrar ☐

Technologist ☐

QUESTIONNAIRE

17 OCTOBER 2018

1) **Q:** What is the maximum allowable time interval between specimen collection and testing for aPTT? (Please do not state a range) (1 mark)

   A: *4 hours*

2) **Q:** Is the transportation of whole blood coagulation specimens on ice acceptable? Explain. (2 marks)

   A: *No. Transportation of whole blood specimens on ice is not recommended for most plasma based coagulation assays. Transportation on ice may result in activation of factor VII, loss of von Willebrand factor and platelet disruption.*

3) **Q:** For coagulation collection tubes, what percentage fill/draw volume is considered underfilled? (1 mark)

   A: *Less than 90%*

4) **Q:** For coagulation collection tubes, what percentage fill/draw volume is considered overfilled? (1 mark)

   A: *More than 110%*

5) **Q:** What procedure should be followed when receiving a specimen for coagulation assays in a patient with a haematocrit > 55%? (1 mark)

   A: *The coagulation specimen with an elevated haematocrit (>55%) results in falsely prolonged clotting times and therefore needs to be rejected. A modified coagulation collection tube has to be prepared where a specific quantity of sodium citrate is removed. The volume of sodium citrate that needs to be removed can be calculated from an equation or normogram chart. The treating clinician needs to be informed and the modified collection tube can be collected from the laboratory for blood sampling.*

6) **Q:** Optimal centrifugation is paramount to ensure platelet poor plasma. What should the platelet count be for a specimen to be considered platelet poor? (1 mark)

   A: *Less than 10 x 10^9/L*

7) **Q:** How often should the centrifugation procedure be validated? (1 mark)

   A: *Every 6 months or after modification of the centrifuge.*

8) **Q:** Please review the graph in addendum A. Explain how you would proceed. (2 marks)

   A: *Verify sample and reagent integrity. Assess for the presence of a blood clot. Visual inspection of the coagulation analyser reaction curve. Repeat measurement. Repeat blood sampling if error persists.*
