# Supplementary material for: The impact of laboratory staff training workshops on coagulation specimen rejection rates
Source: PLoS One. 2022 Jun 3;17(6):e0268764. doi: 10.1371/journal.pone.0268764 (PMC9165799; doi:10.1371/journal.pone.0268764)
Supplement: S9 Appendix — (PDF) [file pone.0268764.s019.pdf]

Standard Operating Procedure (SOP)

Coagulation Laboratory - Tygerberg Academic Hospital

Pre-analytical Variables and Specimen Rejection in accordance with the Clinical Laboratory Standards Institute (CLSI) H21-A5; Vol. 28; No. 5 (2008) & Siemens Sysmex® CS-2100i Coagulation Analyser Guidelines

Rejection Criteria for Coagulation Specimens:

- Coagulation specimens received in collection tubes that contain an additive other than sodium citrate (ex: oxalate, heparin & EDTA).
- Coagulation specimens received in expired collection tubes.
- Coagulation collection tubes that contain 3.8% sodium citrate.
- Unlabelled coagulation specimens.
- Mislabelled coagulation specimens (i.e. a discrepancy between the details on the specimen and the details on the request form).
- Specimens where the collection date and time is NOT stated.
- Whole blood coagulation specimens received on ice.
- Clotted specimens.
- Underfilled coagulation collection tubes (< 90% fill volume in relation to the optimal fill volume).
- Overfilled coagulation collection tubes (> 110% fill volume in relation to the optimal fill volume).
- Coagulation specimens where the haematocrit is greater than 55% (> 0.55 L/L).
- ALL haemolysed specimens.
- Lipemic specimens where no result is generated. (When lipemia is flagged and a result is generated, the result can be authorized with a comment that the sample is lipemic and the result is to be treated with reserve).
- Icteric specimens where no result is generated. (When a sample is flagged as icteric and a result is generated, the result can be authorized with a comment that the sample is icteric and the result is to be treated with reserve).
- For PT assays, specimens older than 24 hours.
- For non-PT assays (ex: aPTT, fibrinogen, D-dimer), specimens older than 4 hours.
- Specimens from heparinized patients for aPTT assays that are not centrifuged within one hour. (Applicable to specimens received in tubes that contain sodium citrate additive).
- Specimens for anti-factor Xa assays received more than one hour after collection.

Created by:

- Dr. M. du Toit (Registrar)..
- Ms. Du Plessis (Senior Technologist)...

Verified by:

- Dr. N. Mashigo (Consultant)...
- Dr. Z.C. Chapanduka (Consultant; HOD)..
