# Supplementary material for: The impact of laboratory staff training workshops on coagulation specimen rejection rates
Source: PLoS One. 2022 Jun 3;17(6):e0268764. doi: 10.1371/journal.pone.0268764 (PMC9165799; doi:10.1371/journal.pone.0268764)
Supplement: S10 Appendix — (PDF) [file pone.0268764.s020.pdf]

3. **FIBRINOGEN ASSAY (Clause Technique)**
   Dilutes plasma is clotted with a strong thrombin solution. . A Calibration curved is prepared each time the thrombin lot number is changed. The calibration curve is use to calculate the results of unknown plasma samples.

4. **THROMBIN TIME**
   Thrombin is added to plasma and the clotting time is measured.

5. **D-DIMER ASSAY**
   Polystyrene particles covalently coated with a monoclonal antibody are aggregated when mixed with samples containing D-Dimer. The D-Dimer cross-linkage region has a stereo symmetrical structure i.e. the epitope for the monoclonal antibody occurs twice. Concequently one antibody suffices to trigger an aggregation reaction, which is then detected turbudimetrically via the increase in turbidity.

**PERFORMING PLATELET CHECK FOR OPTIMUM CENTRIFUGE FUNCTION**

Perform the following procedure once a month. Complete platelet log sheet and file Advia print out in appropriate file

- Centrifuge citrate sample for 15 minutes at 4000rpm
- First run a primer
- Run citrate plasma sample
- A platelet count of < 10 is acceptable
- Complete relevant log sheet
- Print and file results
- A platelet count of >10 should be monitored and if persists, the centrifuge service engineer must be contracted

**ELEVATED HAEMATOCRITS >55.0 L/L (>55%)**

**Formula to Calculate the Volume of Sodium Citrate**

$$C = (1.85 \times 10^{-3})(100-H)V$$

C= Volume of Sodium Citrate in milliliters
H = Patient haematocrit
V = Tube size in milliliters

Reference 4, page 738

**REJECTION CRITERIA FOR COAGULATION STUDIES**

- Reject any sample that has a clot or fibrin strands, no matter how small
- Reject any sample that is under filled - Not within the 10% of the stated volume
- Reject any sample with no identification on the tube and it could not be confirm
- Reject any sample if the specimen is older than 6 hours and not frozen except for INR requests (24 hours)
- Reject any sample that is more than 1+ haemolysed
- Reject any sample that has been drawn in a expired tube
- If the patient hematocrit is >55%, a special tube must be prepared

**RUNNING SAMPLES – AUTOMATIC INQUIRY (Barcoded and Host Query)**

- When the CS-2100i is connected to the host computer using bi-directional communication, no operator intervention is required for registering test parameters
- If the CS-2100i is in the READY mode (green light in front of the instrument ), load the samples into a rack, ensuring the barcode faces the spaces in the rack


*National Health Laboratory Service- All rights reserved*
