# Supplementary material for: The impact of laboratory staff training workshops on coagulation specimen rejection rates
Source: PLoS One. 2022 Jun 3;17(6):e0268764. doi: 10.1371/journal.pone.0268764 (PMC9165799; doi:10.1371/journal.pone.0268764)
Supplement: S11 Appendix — (PDF) [file pone.0268764.s021.pdf]

3. **FIBRINOGEN ASSAY (Clause Technique)**
Dilutes plasma is clotted with a strong thrombin solution. A Calibration curved is prepared each time the thrombin lot number is changed. The calibration curve is use to calculate the results of unknown plasma samples.

4. **THROMBIN TIME**
Thrombin is added to plasma and the clotting time is measured.

5. **D-DIMER ASSAY**
Polystyrene particles covalently coated with a monoclonal antibody are aggregated when mixed with samples containing D-Dimer. The D-Dimer cross-linkage region has a stereo symmetrical structure i.e. the epitope for the monoclonal antibody occurs twice. Concequently one antibody suffices to trigger an aggregation reaction, which is then detected turbudimetrically via the increase in turbidity.

PERFORMING PLATELET CHECK FOR OPTIMUM CENTRIFUGE FUNCTION

Perform the following procedure once a month. Complete platelet log sheet and file Advia print out in appropriate file

- Centrifuge citrate sample for 15 minutes at 4000rpm
- First run a primer
- Run citrate plasma sample
- A platelet count of < 10 is acceptable
- Complete relevant log sheet
- Print and file results
- A platelet count of >10 should be monitored and if persists, the centrifuge service engineer must be contracted

ELEVATED HAEMATOCRITS >55.0 L/L (>55%)

Formula to Calculate the Volume of Sodium Citrate

$$C = (1.85 \times 10^{-3})(100-H)V$$

C= Volume of Sodium Citrate in milliliters
H = Patient haematocrit
V = Tube size in milliliters

Reference 4, page 738

REJECTION CRITERIA FOR COAGULATION STUDIES

- Reject underfilled coagulation collection tubes (< 90% fill volume in relation to the optimal fill volume).
- Reject ALL haemolysed coagulation specimens.
- Reject clotted coagulation specimens.
- For PT assays, reject specimens older than 24 hours.
- For non-PT assays (e.g.: aPTT, fibrinogen, D-dimer), reject specimens older than 4 hours.
- For aPTT assays from heparinized patients, reject specimens that are not centrifuged within one hour.
- Reject specimens for anti-factor Xa assays received more than one hour after specimen collection.
- Reject coagulation specimens received in collection tubes that contain an additive other than sodium citrate (eg: oxalate, heparin & EDTA).
- Reject coagulation specimens received in collection tubes that contain 3.8% sodium citrate.
- Reject coagulation specimens received in expired collection tubes.
- Reject unlabeled coagulation specimens.

In the event of a dispute concerning this document, the electronic version stored on Q-Pulse will be deemed to be the correct version

National Health Laboratory Service- All rights reserved

- Reject mislabeled coagulation specimens (i.e. a discrepancy between the details on the specimen and the details on the request form).
- Reject coagulation specimens where the collection date and time is NOT stated.
- Reject whole blood coagulation specimens AND plasma specimens received on ice.
- Reject overfilled coagulation collection tubes (> 110% fill volume in relation to the optimal fill volume).
- Reject coagulation specimens where the haematocrit is greater than 55% (> 0.55 L/L).
- Reject lipaemic specimens where no result is generated. (When lipaemia is flagged and a result is generated, the result can be authorized with a comment that the sample is lipaemic and the result is to be treated with reserve).
- Reject icteric specimens where no result is generated. (When a sample is flagged as icteric and a result is generated, the result can be authorized with a comment that the sample is icteric and the result is to be treated with reserve).

RUNNING SAMPLES – AUTOMATIC INQUIRY (Barcoded and Host Query)

- When the CS-2100i is connected to the host computer using bi-directional communication, no operator intervention is required for registering test parameters
- If the CS-2100i is in the READY mode (green light in front of the instrument), load the samples into a rack, ensuring the barcode faces the spaces in the rack
- Place the loaded rack in the right side input area (Make sure the rack is correctly placed, barcode facing the instrument)
- Press start – the rack will automatically be moved forward and cross the front of the instrument where the sample will be aspirated
- When the ID is read, the host query takes place and the test parameters are automatically requested
- Additional racks can be added to the right input area while the instrument is busy
- Press Start button after loading a new rack
- Remove the rack from the left side when completed

RUNNING SAMPLES – MANUAL ORDERING OF TESTS

- From the main screen select **ORDER**
- Enter the rack barcode number
- Select **ORDER ENTRY** on the right side of the screen
- Enter sample / lab number for sample position 1
- Press **ENTER** on **Order Entry Screen**
- Press parameter keys to order Fibrinogen test
- Press down error to move cursor to next position
- Continue ordering of all samples to be processed
- Press **OK** on **Order Entry Screen**
- Press **SAVE**
- Press **START**.... processing will begin

RUNNING SAMPLES – MICRO-MODE (Use for pediatric samples and IQC)

- From the main screen select **ORDER**
- Enter the rack barcode number

National Health Laboratory Service- All rights reserved
